# Feasibility of a Sensor-Controlled Digital Game for Heart Failure Self-management: Randomized Controlled Trial

**DOI:** 10.2196/29044

**Published:** 2021-11-08

**Authors:** Kavita Radhakrishnan, Christine Julien, Tom Baranowski, Matthew O'Hair, Grace Lee, Atami Sagna De Main, Catherine Allen, Bindu Viswanathan, Edison Thomaz, Miyong Kim

**Affiliations:** 1 School of Nursing The University of Texas Austin Austin, TX United States; 2 Department of Electrical and Computer Engineering Cockrell School of Engineering The University of Texas Austin Austin, TX United States; 3 Baylor College of Medicine Houston, TX United States; 4 Good Life Games, Inc Austin, TX United States; 5 Department of Statistics and Data Sciences The University of Texas Austin Austin, TX United States

**Keywords:** heart failure, digital game, sensor, self-management, older adults, weight monitoring, physical activity, behaviors, mobile phone

## Abstract

**Background:**

Poor self-management of heart failure (HF) contributes to devastating health consequences. Our innovative sensor-controlled digital game (SCDG) integrates data from sensors to trigger game rewards, progress, and feedback based on the real-time behaviors of individuals with HF.

**Objective:**

The aim of this study is to compare daily weight monitoring and physical activity behavior adherence by older adults using an SCDG intervention versus a sensors-only intervention in a feasibility randomized controlled trial.

**Methods:**

English-speaking adults with HF aged 55 years or older who owned a smartphone and could walk unassisted were recruited from Texas and Oklahoma from November 2019 to August 2020. Both groups were given activity trackers and smart weighing scales to track behaviors for 12 weeks. The feasibility outcomes of recruitment, retention, intervention engagement, and satisfaction were assessed. In addition to daily weight monitoring and physical activity adherence, the participants’ knowledge, functional status, quality of life, self-reported HF behaviors, motivation to engage in behaviors, and HF-related hospitalization were also compared between the groups at baseline and at 6, 12, and 24 weeks.

**Results:**

A total of 38 participants with HF—intervention group (IG; 19/38, 50%) and control group (CG; 19/38, 50%)—were enrolled in the study. Of the 38 participants, 18 (47%) were women, 18 (47%) were aged 65 years or older, 21 (55%) had been hospitalized with HF in the past 6 months, and 29 (76%) were White. Furthermore, of these 38 participants, 31 (82%)—IG (15/19, 79%) and CG (16/19, 84%)—had both weight monitoring and physical activity data at the end of 12 weeks, and 27 (71%)—IG (14/19, 74%) and CG (13/19, 68%)—participated in follow-up assessments at 24 weeks. For the IG participants who installed the SCDG app (15/19, 79%), the number of days each player opened the game app was strongly associated with the number of days the player engaged in weight monitoring (*r*=0.72; *P=*.04) and the number of days with physical activity step data (*r*=0.9; *P*<.001). The IG participants who completed the satisfaction survey (13/19, 68%) reported that the SCDG was easy to use. Trends of improvement in daily weight monitoring and physical activity in the IG, as well as within-group improvements in HF functional status, quality of life, knowledge, self-efficacy, and HF hospitalization in both groups, were observed in this feasibility trial.

**Conclusions:**

Playing an SCDG on smartphones was feasible and acceptable for older adults with HF for motivating daily weight monitoring and physical activity. A larger efficacy trial of the SCDG intervention will be needed to validate trends of improvement in daily weight monitoring and physical activity behaviors.

**Trial Registration:**

ClinicalTrials.gov NCT03947983; https://clinicaltrials.gov/ct2/show/NCT03947983

## Introduction

### Need for Heart Failure Self-management Behaviors

Despite significant advances in treatment and management, heart failure (HF) continues to be the leading cause of hospitalization among older adults in the United States [1,2], with an estimated annual cost of US $32 billion [1]. Improved self-management behaviors, defined as “behaviors that maintain physiological stability” and enable “response to symptoms when they occur,” can help reduce the adverse effects of an HF diagnosis [3,4]. Daily weight monitoring is such a behavior; weight gain is typically the first sign of volume overload in patients with HF, and if weight gain is treated promptly, clinically significant HF exacerbations can be avoided [5,6]. Similarly, physical activity improves myocardial function and functional capacity [7] and reduces depressive symptoms [8]. Engagement in these 2 critical HF self-management behaviors improves quality of life and reduces health care use [9,10]. Yet, weight monitoring and physical activity show significantly poorer adherence than other HF self-management behaviors [11,12] because of poor knowledge and lack of motivation [13].

### Role of Innovative Digital Health Interventions for Motivating HF Self-management Behaviors

Recent advances in wearable technology, digital sensor devices, and mobile health (mHealth) apps have allowed application of these technologies to observe and motivate health behaviors in real-world conditions. Yet, despite the advantages of portability and scalability offered by digital devices and apps, long-term adoption rates remain low. There have been many instances of abandonment of the use of activity trackers in prior research, with more than 50% of the activity tracker users stopping use within a period of 2 weeks to 6 months [14,15]. Long-term maintenance rates of such devices by patients with HF are yet to be explored.

Digital games that serve as affordable, portable, scalable tools while being enjoyable and easy to use can help contextualize health behaviors [16] and motivate clinically significant behavior changes, thereby improving health outcomes [17]. Digital games can combine appealing stories [18] and interests and hobbies [19] with active learning [20], incentives [21], and social connections [22] to offer an accessible, engaging, and immersive habit-forming medium [23] while objectively measuring these behaviors [24,25]. Well-designed digital games have significantly improved physical (eg, balance and mobility) and cognitive (eg, processing speed) health outcomes [26] as well as behavioral outcomes for chronic diseases (eg, dietary changes for diabetes) [17,27]. Digital game-playing is increasing among older adults; the number of adults aged 50 years or older who enjoy video games in any format increased by 11 million from 2016 to 2019 [28], suggesting a receptiveness to playing games for health.

Digital games incorporating gamification principles such as competition, leaderboards, and incentives have improved learning outcomes and have been highly acceptable among participants with HF [29,30], but these games do not involve data on real-time HF self-management behaviors. In a sensor-controlled digital game (SCDG), data on behaviors from sensors, including those in wearable devices, are synchronized with a mobile gaming app to trigger game progress, rewards, personalized and contextually relevant feedback (eg, reduce fluid intake or call physician for weight gain), and incentives based on participants’ real-time behaviors [24]. Combining digital games and sensors thus offers a powerful way to improve behavior adherence and potentially make health care participatory, personalized, predictive, and preventive as defined by the Precision Medicine Initiative [31].

Thus, the primary goals of this study are to obtain preliminary efficacy data on behavior adherence and undertake a comprehensive feasibility assessment of an SCDG intervention called Heart Health Mountain. This app synchronizes with a Bluetooth-enabled weighing scale and activity tracker to activate game rewards and feedback based on the real-time weight monitoring and exercise behaviors of older adult participants with HF.

## Methods

### Study Design and Scope

We conducted a prospective feasibility randomized controlled trial (1:1) with 2 parallel groups (sensors only or sensors plus SCDG app) from November 2019 to January 2021 (ClinicalTrials.gov: NCT03947983). The results presented here are from the 12-week SCDG intervention measuring objective behaviors of daily weight monitoring and physical activity and the 24-week follow-up measuring quality of life, functional status, HF self-management efficacy, and HF hospitalizations.

### Study Population and Recruitment

Before the COVID-19 outbreak, we identified potential participants through chart review at a cardiac rehabilitation center as well as through referrals by clinical case managers at an inpatient cardiac floor in central Texas. Research staff members provided information on the study to adults who were aged 55 years or older, English-speaking, diagnosed with HF, and classified according to the New York Heart Association’s HF classification as class II or III [32] during their inpatient stay or outpatient visit to the cardiac center. Other eligibility criteria included smartphone ownership as a proxy measure to indicate prior familiarity with smartphone use, ability to independently walk without a walker or human assistance, and a score of 4 or higher on the Mini-Cog [33] cognitive screen. The exclusion criteria included severe visual or tactile impairments (eg, legal blindness or severe arthritis), which would prevent the use of a smartphone, or end-stage renal failure or terminal illness (eg, cancer), both of which adversely affect HF prognosis [34].

To continue the trial during the COVID-19 pandemic, all in-person interactions were converted to remote interactions. We used a secure, Health Insurance Portability and Accountability Act–compliant email system to continue receiving referrals from clinical case managers at the inpatient HF center. In addition, we contracted with the participant recruitment company Trialfacts [35] to recruit participants on the web from the states of Texas and Oklahoma. The eligibility criteria remained the same, but the formal screening process then required a narrative description of HF history or confirmation from the potential participant’s health care provider to confirm the individual’s HF diagnosis.

### Intervention

The description of the design, development, and usability assessment phase of the SCDG has been detailed elsewhere [25]. Guided by the Fogg behavioral model [36] and played on a smartphone, the *Heart Health Mountain* SCDG presented a narrative in which the older adult player helps an avatar to climb a mountain in a forested area, with the game’s goal being to avoid hospitalization. The SCDG motivates the player’s engagement in critical behaviors related to HF self-management, helping the avatar to climb the mountain. This is done by daily weight monitoring and attaining physical activity steps based on goal steps tailored to the individual player. The step goal in the SCDG ranged from 3000 to 15,000 steps.

**Figure 1:**
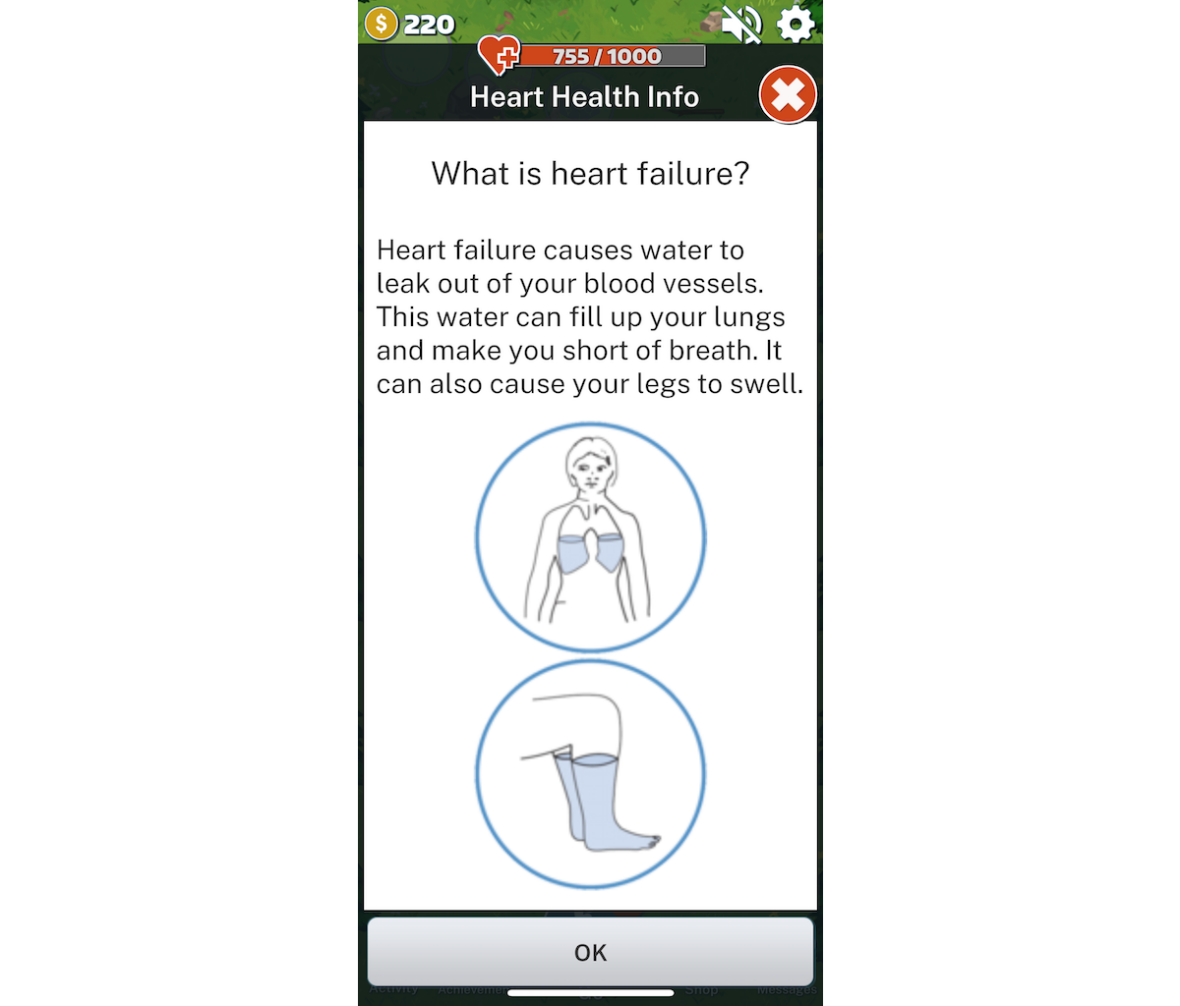
Knowledge embedded in the sensor-controlled digital game.

**Figure 2:**
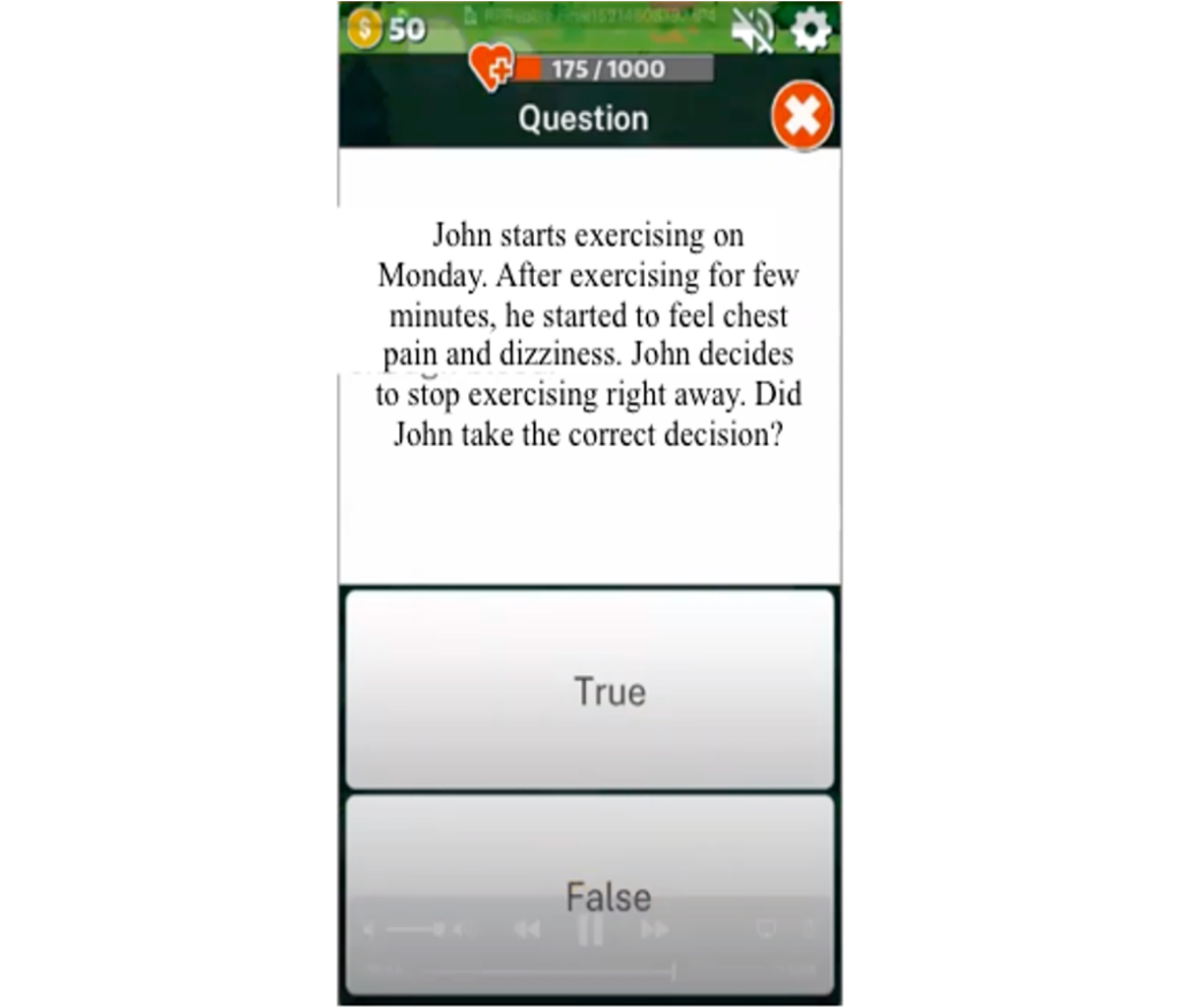
Built-in quiz in the sensor-controlled digital game.

According to the Fogg model [36], for a person to perform a target health behavior, they must (1) be sufficiently *motivated*, (2) have the *ability* to perform the behavior, and (3) be *triggered* to perform the behavior. For example, based on the *motivation* concept of the Fogg model [36], positive attitudes toward engaging in HF self-management and empathy with the game character will be aided by positive feedback, competition (eg, leaderboards), and game rewards (eg, coins to buy low-salt food items or accessories for the game character for engaging in real-time behaviors and in-game challenges). On the basis of the *ability* concept of the Fogg model [36], knowledge about HF self-management within the SCDG was provided using language especially chosen for those with low literacy levels (from the *Living Well With Heart Failure* booklet) [37,38] in bite-sized chunks (Figure 1). Built-in quizzes tested content mastery, and problem-solving strategies provided opportunities for higher game rewards (Figure 2).

**Figure 3:**
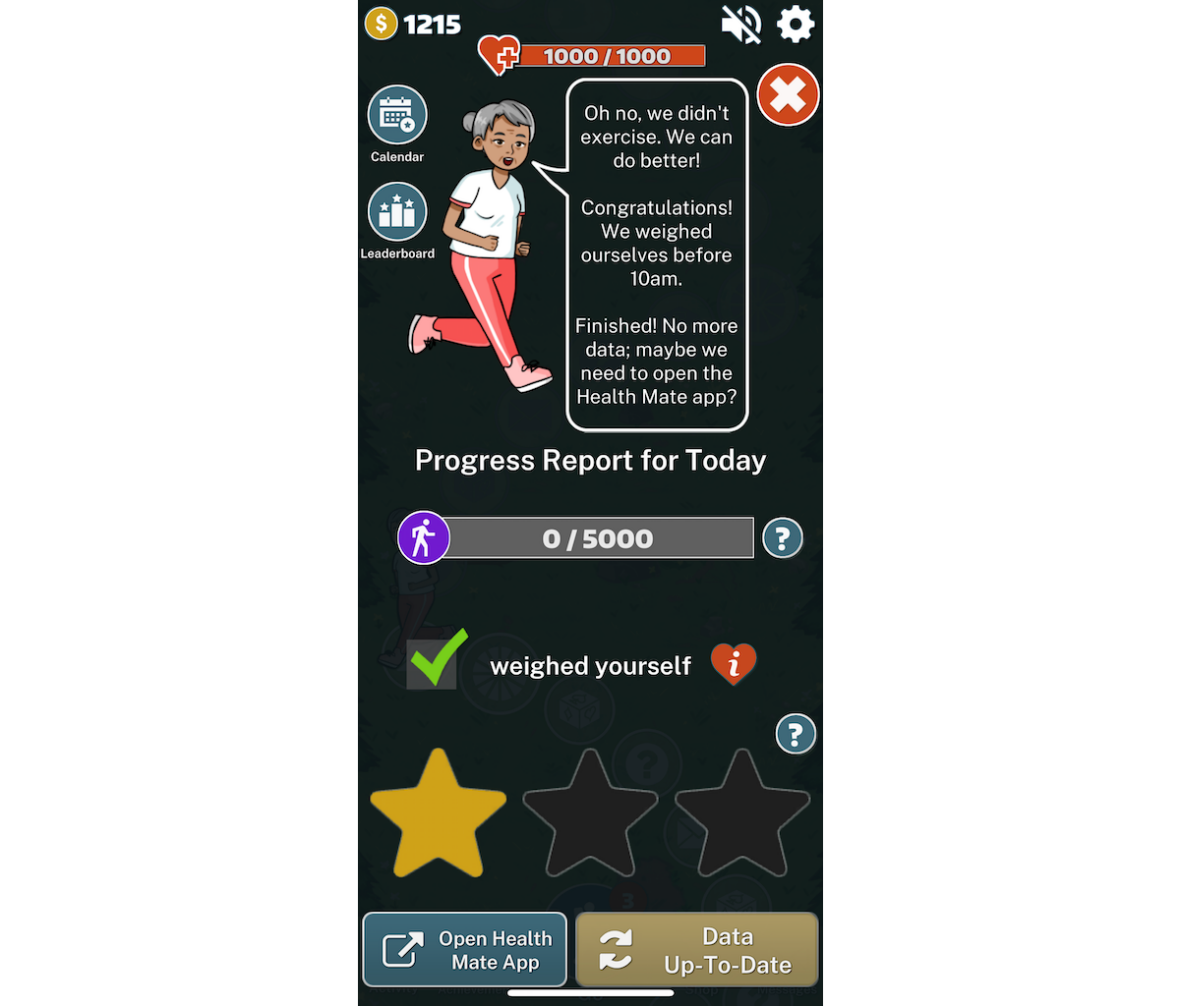
Example of game alert messages based on real-time behavior.

Finally, based on the *trigger* concept of the Fogg model [36], game alerts, the avatar’s health status, messages (Figure 3), and incentives were tailored to the participant’s real-time HF self-management behaviors based on data from the behavior-tracking sensors (eg, climbing up the mountain by 2 steps if the physical activity goal was attained that day). HF game players are expected to make meaningful connections between game events and real-time HF self-management behaviors, increasing the likelihood that they retain and apply their newly acquired knowledge, skills, and habits for HF self-management behaviors outside the game’s world [39] and improve their health outcomes.

### Intervention Procedures

CONSORT (Consolidated Standards of Reporting Trials) guidelines [40] informed this parallel-group randomized controlled trial and the reporting of outcomes (Multimedia Appendix 1). The SCDG intervention group (IG) received sensors tracking weight monitoring and physical activity and played the SCDG app on a smartphone; the sensors-only control group (CG) received sensors tracking weight monitoring and physical activity only. Both IG and CG participants were given the Withings Go activity tracker (Withings) [41], Withings Body smart weighing scale [42], and Withings Health Mate app [43] to record, store, and transmit daily weight and physical activity data, but the CG did not receive the SCDG app. The Withings Body smart weighing scales have been found to accurately measure body mass [44]. We selected the Withings Go tracker because it has long battery life, is waterproof, is reasonably accurate in measuring activity levels of people with HF [45,46], and uses a clock schema, which is a familiar interface for most adults, to represent the user’s daily physical activity. The step goals for all participants were set based on their preferences as well as the nursing research assistant’s assessment of their physical health status.

Whereas the participants in the IG received standardized HF education [6,38] embedded within the SCDG, the participants in the CG received the same information in written format. Thus, the difference between the IG and the CG was the receipt of the SCDG gaming elements. The SCDG app transmitted the IG participants’ game-playing data, and the Health Mate app transmitted all participants’ sensor data to the research team through cellular data service or home Wi-Fi. An intervention nursing research assistant remotely tracked user engagement for participants in both groups during the first week and contacted participants by phone in the absence of any data to offer help with troubleshooting.

Before the COVID-19 outbreak, an intervention nursing research assistant installed the apps on all participants’ smartphones and trained the participants to use the sensor devices and apps at either their homes or at a location of their convenience. During the COVID-19 pandemic, in-person interactions were converted to remote interactions. Contactless delivery of study equipment, videoconferencing on smartphones, and printed pictorial and video instructions allowed remote support for installation, training, and troubleshooting of the devices and apps. The study team members’ experience with the devices and apps during in-person interactions with the older adults at their homes helped inform the training materials that were provided to the participants during the pandemic. Installation and training times by the research team member varied widely during the remote phase, from 0 minutes (participant self-installation) to 180 minutes compared with 45 to 120 minutes during the in-person phase.

### Randomization

The participants were assigned to the IG or CG with a 1:1 randomization ratio such that the 2 groups were equivalent in terms of biological sex. The randomization was carried out after informed consent was obtained and the baseline survey completed. The allocation sequence list for block randomization was generated by an independent researcher at the Sealed Envelope Ltd website [47] with random block sizes of 2 and 4 and concealed until the trial group was assigned. To ensure blinding during assessment of the outcomes, the research assistant who delivered the intervention to the 2 groups was different from the research assistant who collected the baseline and follow-up surveys from the 2 groups. The researcher who performed the data analysis was blinded to the participant groups. Indication of participation in a digital health intervention study in the informed consent allowed us to blind all participants to their specific group assignment.

### Outcome Measures

#### Feasibility Outcomes

To inform the development of a future large-scale randomized controlled clinical trial, we determined the feasibility, acceptability, and effectiveness of our SCDG in engaging older adults with HF by assessing recruitment (how many accepted the invitation to participate in the study), retention, engagement, and satisfaction. We considered the trial feasible if the enrollment rate was at least 20% of the patients with HF who were approached to participate in the study, if at least 50% of the IG and CG participants completed both the 12-week objective behavior assessment and the 24-week follow-up assessment (retention), and if at least 50% of the IG participants used the SCDG intervention for at least 50% of the days (engagement) and were satisfied with the SCDG intervention (satisfaction).

#### Retention

Retention was recorded as the number (proportion) of participants in both groups who used the sensor devices to measure physical activity and weight monitoring for 12 weeks and completed follow-up assessments at 24 weeks to assess maintenance of the SCDG intervention.

#### Engagement and Adherence

A Google Cloud console was created to store all participants’ deidentified physical activity and weight monitoring behavioral data obtained from the Withings Health Mate app as well as data used to assess the IG participants’ engagement with the SCDG and its features. The number of days of adherence to physical activity and weight monitoring as well as engagement with the SCDG were obtained from the Google Cloud console.

#### Satisfaction

We assessed the IG participants’ satisfaction with the SCDG using a questionnaire at the end of 12 weeks based on the 4-item interest and enjoyment subscale of the Intrinsic Motivation Inventory (α=.8) [48], a multidimensional instrument intended to assess participants’ subjective experience related to a target activity. The interest and enjoyment subscale assesses an individual’s interest and inherent pleasure in performing a specific activity on a 4-point Likert scale (1=not satisfied, 4=very satisfied). In addition, the participants were asked open-ended questions about what they liked and disliked most about using the SCDG for HF self-management. A 4-point scale (1=strongly disagree, 4=strongly agree) was used to assess the key elements that might have helped the participants maintain their motivation to continue using the SCDG. Finally, the participants were asked whether they would recommend the SCDG to others with HF.

### Health and Behavior Outcomes

#### Overview

All participants were asked to complete surveys at 6, 12, and 24 weeks after the baseline survey to assess the immediate effect of the intervention and maintenance of behavioral changes. Before the COVID-19 outbreak, the participants were asked to complete the surveys on the Qualtrics platform (Qualtrics XM) on a study iPad (Apple Inc). During the pandemic, the participants were emailed or texted links to the Qualtrics surveys. All participants completed a sociodemographic survey questionnaire at baseline. They also completed the 2-item Patient Health Questionnaire [49] to report on depressive symptoms at baseline and at 6, 12, and 24 weeks.

#### Weight Monitoring Behavior

The primary outcome of the days of weight monitoring was measured by the number of days with weight monitoring data. This measure was collected from sensor logs within the Health Mate app [43], with each day of weighing measured dichotomously (yes or no).

#### Physical Activity Behavior

Physical activity data were derived from the Withings Go sensor logs within the Health Mate app [43] by obtaining the cumulative steps for each day and averaging the steps for each participant over 6 and 12 weeks.

#### HF-Related Functional Status

For this measure, we used items 1-12 from the Kansas City Cardiomyopathy Questionnaire (KCCQ) [50]. The item scores are transformed to a range of 0 to 100 by subtracting the lowest possible scale score, dividing by the range of the scale, and multiplying by 100. The summary scores for the functional status thus range from 0 to 100, with higher scores indicating better functional status. The Cronbach α value for the KCCQ functional status is excellent at .93 [49]. The scale has demonstrated criterion validity, with high correlation with the New York Heart Association HF classification and the 6-minute Walk Test [50].

#### Quality of Life

Quality of life was measured with items 13-15 from the KCCQ [50]. The item scores are transformed to a range of 0 to 100 by subtracting the lowest possible scale score, dividing by the range of the scale, and multiplying by 100. The summary values for the quality-of-life domain thus range from 0 to 100, with higher scores indicating better quality of life. The Cronbach α value was .78 in prior studies [50]. In comparison with similar quality-of-life instruments, the KCCQ is sensitive to clinical changes in HF and has shown a significant and high correlation with the New York Heart Association HF classification [50].

#### HF Self-management Knowledge

The instrument used was the 30-item Atlanta Heart Failure Knowledge Test [51]. Each correct answer is scored as 1, with no additional weighting of items; the correct responses are then summed. Incorrect or skipped questions are scored as 0. The total scores range from 0 to 30, and higher scores indicate better HF knowledge. The content validity ratings for relevance and clarity ranged from 0.55 to 1.0, with 81% of the items rated 0.88 to 1.0. The Cronbach α value for reliability was .84 [51]. Construct validity has been demonstrated by directly correlating knowledge with clinical and self-care outcomes, including dietary sodium consumption, medication adherence, and health care use [51].

#### Self-reported HF Self-care Behaviors

The instrument used was the 9-item European Heart Failure Self-care Behavior Scale [52]. The items were scored on a Likert scale of 1 to 5. For calculating the standardized score, each item was reverse coded and then computed using the following formula: (sum of all even-coded items minus 9) times 2.7777. The standardized scores range from 0 to 100; every item is given equal weight, with a higher score indicating better self-care. The coefficient α value was .80 for this instrument [52].

#### HF Self-efficacy

The instrument used was the 6-item Section C (self-efficacy section) of the Self-Care of Heart Failure Index [53]. The item scores were standardized using the following formula: (sum of Section C items minus 6) times 5.56. The scores on the self-efficacy scale range from 0 to 100, with higher scores reflecting better self-efficacy. The Cronbach α value was .88, with good evidence for construct validity and contrasting group validity [53].

#### Motivation for HF Self-management Behaviors

The instrument used was the 19-item Treatment Self-Regulation Questionnaire [54], adapted for HF. Each item was scored on a scale of 1 to 7. The scale has 2 subscales: autonomous regulation and controlled regulation. The score for each subscale was the average of the items in that subscale. The average for controlled regulation was subtracted from the average for autonomous regulation to calculate the Relative Autonomy Index. The Cronbach α value for chronic disease–related behaviors ranged from .83 to .87 [54]. Construct and convergent validity have been confirmed using factor analysis and correlations with autonomy-related subscales of other behavior scales and health outcomes [55].

#### HF Hospitalization

This measure was obtained through the participants’ self-report (yes or no) on the 6-, 12-, and 24-week surveys and was confirmed with hospitalization discharge summaries or communication with health care providers.

### Statistical Analysis

#### Power

On the basis of 1:1 randomization with 80% power (α=5%), a sample size of 38 patients per group would have been required to detect a difference of 80% versus 50% in both groups of daily monitoring of weight monitoring and physical activity. We chose 80% as the cutoff for adequate weight monitoring at 12 weeks because patients with HF who completed *at least* 80% of the weight diaries (5.6/7 of the days per week) were found to have significantly reduced odds for HF-related hospitalizations in comparison with patients who completed less than 80% of the weight diaries [6]. In addition, only 50% of the participants with HF in a remote monitoring sensor group recorded their weights more than 50% of the time [56]. Allowing for 10% attrition, 49 patients per group (N=98) would have had to be recruited for a fully powered clinical trial of the SCDG intervention for HF behavior of weight monitoring. The power analysis was performed using G*Power (Heinrich Heine University) [57]. With a sample of 38, our study was underpowered. However, this sample size was sufficient to conduct a feasibility study [58], which can inform implementation of a fully powered study with fewer problems to test the SCDG’s effectiveness.

#### Data Analysis

Descriptive statistics for feasibility included (1) the percentage of participants recruited from the total who were approached, (2) the percentage of participants (among those recruited) who were retained in the study at the end of 24 weeks in each arm and overall, (3) the number of days the IG participants played the SCDG, and (4) average satisfaction with the SCDG. All open-ended questions were coded by 2 team members (CA and AS) and then analyzed using a general inductive thematic approach [59].

The observed effect sizes (Cohen *d*) for the primary outcome of weight monitoring days at the end of 12 weeks was calculated using the following formula: mean of days in IG minus mean of days in CG divided by average SD of days in IG and days in CG. The potential clinical meaningfulness of the results (in addition to statistical significance) was based on the magnitude of the effects: small (Cohen *d*=0.20), medium (Cohen *d*=0.50), and large (Cohen *d*=0.80) [60].

All statistical analyses were conducted using SPSS software (version 26.0; IBM Corp). Baseline characteristics of the participants in the IG and CG were compared using independent 2-tailed *t* tests for continuous variables and chi-square tests for categorical variables. Although this study was a feasibility trial, we assessed within-group trends in the IG and CG using paired-sample *t* tests (2-tailed) at baseline, 6 weeks, 12 weeks, and 24 weeks. Within-group changes were presented as absolute changes from baseline. All data were presented as means with SDs. Missing values were addressed using intent-to-treat principles with maximum likelihood estimations.

### Ethical Considerations

The institutional review board at the University of Texas at Austin approved this study on July 30, 2018 (number 2017-12-0042). Interested participants provided their phone numbers to be contacted by a nursing graduate research assistant for formal screening through phone. If potential participants were found eligible, the research assistant scheduled visits at their homes to complete the written informed consent.

During the COVID-19 pandemic, the eligible potential participants completed the verbal informed consent with the research assistant on the phone. The participants were also emailed a copy of the informed consent form. To protect the participants’ privacy, 6-digit unique ID numbers and dummy email addresses were generated for the profile information that was required by the Health Mate app [43] and Google Cloud console so that no personal patient information was shared or processed through the app or the console. Participants who scored higher than 4 on the Patient Health Questionnaire (2 items) for depression [49] were provided resources for local mental health services, including available mental health services at their HF clinical site. The eligible participants received US $75 as incentives spread over 4 time points for participating in the study. The participants who continued in the study during the COVID-19 pandemic were also allowed to keep the weighing scale and tracker to avoid reuse of the devices.

## Results

### Baseline Characteristics

The characteristics of the participants are presented in Table 1. There were no marked differences between the IG and the CG except for marital status, with significantly more IG participants being married or having a partner.

**Table 1:** Baseline characteristics of the cohort (N=38).

Baseline characteristics		SCDG^a^ (IG^b^; n=19), n (%)	Sensors only (CG^c^; n=19), n (%)	Total, n (%)	*P* value	
**Age (years)**						.20
	55-64	11 (58)	9 (47)	20 (53)		
	65-74	7 (37)	5 (26)	12 (32)		
	≥75	1 (5)	5 (26)	6 (15)		
**Sex**						.99
	Male	10 (53)	10 (53)	20 (53)		
	Female	9 (47)	9 (47)	18 (47)		
**Ethnicity**						.07
	Hispanic or Latino	3 (16)	0 (0)	3 (8)		
	Non-Hispanic or Latino	16 (84)	19 (100)	35 (92)		
**Race**						.25
	White	16 (84)	13 (69)	29 (76)		
	African American	2 (11)	4 (21)	6 (16)		
	Native American	0 (0)	1 (5)	1 (3)		
	Other	1 (5)	1 (5)	2 (5)		
**Highest level of education**						.90
	High school	4 (21)	3(16)	7 (18)		
	University education	8 (42)	8 (42)	16 (42)		
	Technical diploma	7 (37)	8 (42)	15 (40)		
**Marital status**						.05
	Married or has a partner	13 (68)	7 (37)	20 (53)		
	Divorced or widowed	6 (32)	12 (63)	18 (47)		
**Living arrangement**						.35
	Living alone	5 (26)	6 (32)	11 (29)		
	Living with others	12 (63)	12 (68)	24 (63)		
	Other	2 (11)	0 (0)	2 (5)		
**Duration with HF^d^ diagnosis**						.63
	<6 months	6 (32)	5 (26)	11 (29)		
	7 months to 1 year	2 (11)	3 (16)	5 (13)		
	1-5 years	7 (37)	5 (26)	12 (32)		
	>5 years	4 (21)	6 (32)	10 (26)		
**Last** **HF** **hospitalization**						.51
	<1 month	5 (26)	6 (32)	11 (29)		
	1-6 months	7 (37)	3 (16)	10 (26)		
	7 months to 1 year	1 (5)	2 (11)	3 (8)		
	>1 year	6 (32)	8 (42)	14 (37)		
**Prior digital game-playing**						.23
	Yes	17 (90)	13 (68)	30 (79)		
	No	2 (10)	6 (32)	8 (21)		
**Depression (>2 on PHQ^e^ -2)**						.99
	No	14 (74)	14 (74)	28 (74)		
	Yes	5 (26)	5 (26)	10 (26)		
**Number of comorbid conditions**						.62
	0-2	12 (63)	13 (68)	25 (66)		
	>2	8 (42)	6 (32)	14 (37)		
**Top 2 comorbid conditions**						
	Hypertension	10 (53)	10 (53)	20 (53)	.99	
	Diabetes	8 (42)	5 (26)	13 (34)	.55	

^a^SCDG: sensor-controlled digital game.

^b^IG: intervention group.

^c^CG: control group.

^d^HF: heart failure.

^e^PHQ: Patient Health Questionnaire.

### Feasibility Outcomes

#### Recruitment and Enrollment

Before the COVID-19 outbreak, 91 older adults with HF were approached at clinical centers (cardiac rehabilitation center, cardiac hospital unit, and senior centers), of whom 25 (27%) were found ineligible for the study. The reasons for exclusion were as follows: not owning smartphones (19/25, 76%), moderate cognitive impairment (2/25, 8%), moving to another state (pre–COVID-19; 2/25, 8%), or not fluent in English (2/25, 8%). Of the 66 eligible participants, 14 (21%) enrolled in the study.

**Figure 4:**
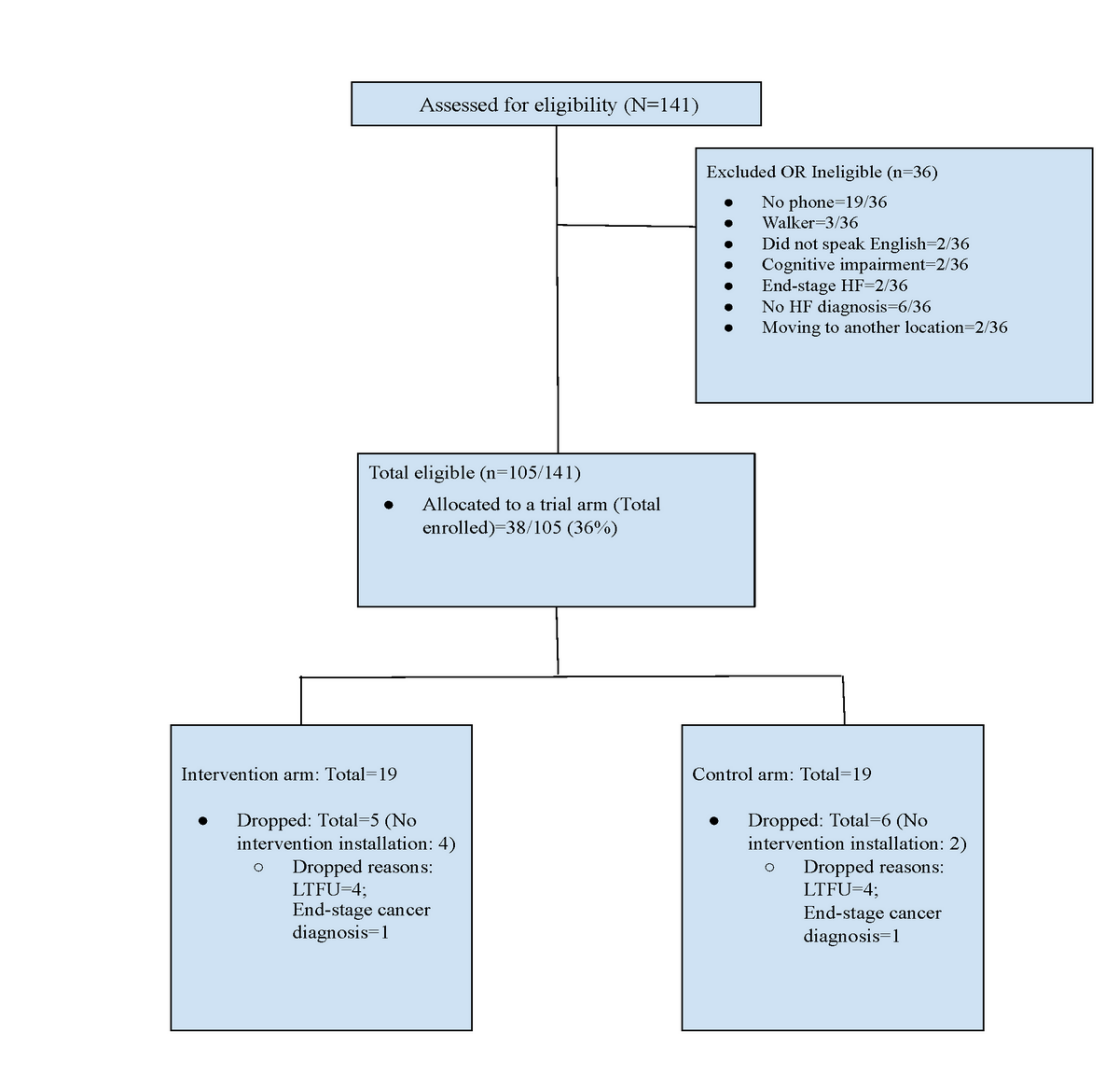
Diagram of participant flow. HF: heart failure; LTFU: lost to follow-up.

During the COVID-19 pandemic, 50 patients with HF were approached, of whom 11 (22%) were ineligible. The reasons for exclusion were as follows: moving to a hospice (2/11, 18%), not having a confirmed diagnosis of HF (6/11, 55%), or using a wheelchair or walker to ambulate (3/11, 27%). Of the 39 participants found eligible—11 (28%) from clinical sites and 28 (72%) through web-based recruitment—24 (61%) agreed to enroll in the study: 6 (25%) from clinical sites and 18 (75%) through web-based recruitment (Figure 4). The average distance of the participants’ homes from the study team increased from 14 miles (range 2-52) during the pre–COVID-19 phase to 143 miles (range 11-465) during the COVID-19 phase.

#### Retention

Of the 38 participants who enrolled in the study, 6 (16%) completed the baseline survey but dropped out before installation of the devices or apps. Of the 6 participants who dropped out, 4 (67%) were lost to follow-up, and 2 dropped out because of end-stage disease unrelated to HF. We obtained the 12-week objective behavior data assessments from 84% (32/38) and 79% (30/38) of the total enrolled participants for weight monitoring and physical activity, respectively. Although 100% (32/32) of the participants who installed the apps were able to transmit data on daily weight monitoring, 6% (2/32; 1 in the CG and 1 in the IG) experienced issues with synchronizing the tracker and were unable to transmit data on physical activity. As the tracker became an outdated version during the course of the study, the device company was unable to support troubleshooting for these 2 trackers. Table 2 provides retention statistics on the survey data available to the study team at each data collection time point.

**Table 2:** Retention of participants at each time point (N=38).

Data collection	Participants, n (%)
12-week weight monitoring	32 (84)
12-week physical activity	30 (79)
6-week surveys	30 (79)
12-week surveys	30 (79)
24-week surveys	27 (71)

#### Engagement

Installation and training for the devices and apps was completed with 100% (32/32) of the participants who progressed in the study. Of the 32 participants, 14 (44%) owned a smartphone with the Android platform, whereas the remaining 18 (56%) owned a smartphone with the iOS platform. Of the 15 remaining IG participants, 11 (71%) played the SCDG more than 50% of the days (range 7%-96%). Using the daily data of the 15 IG participants, the number of days each player opened the game app was strongly associated with the number of days each player engaged in weighing (*r*=0.72; *P=*.04) and the number of days with physical activity step data (*r*=0.9; *P*<.001).

#### Satisfaction With the SCDG

At the end of 12 weeks of game-playing, of the 15 IG participants, 13 (87%) completed the survey regarding their satisfaction with the SCDG (Table 3).

**Table 3:** Intervention group participants’ satisfaction with the sensor-controlled digital game (N=13).

Satisfaction parameters	Participants, n (%)
Interesting	11 (85)
Easy	13 (100)
Enjoyable	11 (85)
Satisfying to play	11 (85)
Satisfaction with the avatar’s look	11 (85)
Satisfaction with sound in the game	12 (92)
Satisfaction with graphics	12 (92)
Satisfaction with using the sensor devices to progress in the game	8 (62)
Satisfaction with the content or information	13 (100)
Game motivated me to weigh myself daily	12 (92)
Game motivated me to exercise more	11 (85)
Will recommend this game to others with HF^a^	11 (85)
Prefer playing digital games over other ways to learn about managing HF	10 (77)

^a^HF: heart failure.

The themes related to playing the SCDG included its competitive nature (2/13, 15%), motivation to attain health behavior goals (5/13, 38%), ease of the interface between the sensor devices and the game (2/13, 15%), opportunity to learn about managing HF (2/13, 15%), and access to behavior data (1/13, 8%). A participant said that the duration of the game play for 12 weeks was not long enough to develop healthy habits:

I was disappointed that it ended so soon. It was motivational for me to continue to pay attention to my heart failure regimen until it became more of a routine. I’m at a stage in my life where I can continue to strengthen my heart by adding more to my routine.

Participant

We faced challenges in syncing data from some of the trackers within the SCDG in real time because the device company discontinued support by the time the study was implemented. Therefore, a common theme among the barriers to playing the game included problems with syncing steps from the Withings Go activity tracker with the game (5/13, 38%). Other barriers included inaccuracies in tracking bicycle activity as steps and the lowest attainable step goal in the game being too high for a participant with HF:

Steps and physical activity at unrealistic start levels. I started out at 280 steps, got up to 550 steps a day.

Participant

Of the 13 participants who completed the survey, 2 (15%) found the game to be simplistic and said they would prefer more features. A participant stated as follows:

Needs more bells and whistles. More color and animations.

Participant

Finally, of the 13 participants who completed the survey, 3 (23%) stated that the use of digital games to motivate HF behaviors was not appealing. Of these 3 participants, 2 (67%) preferred reading to playing games, whereas 1 (33%) stated as follows:

If it would help people do daily weight/vital sign checks it would be worthwhile. I didn’t need motivation other than my cardiologist telling me to.

Participant

### Health and Behavior Outcomes

#### Weight Monitoring

At the end of 12 weeks, in comparison with baseline self-reports of weighing behaviors, trends of increase by 40% in weighing 5 days or more in a week were observed in the IG. Trends of decrease by 6% were observed in the CG for weighing 5 days or more in a week (Table 4). An effect size of 0.53 for the SCDG intervention was obtained for the primary outcome of mean days with weight monitoring.

**Table 4:** Behavior outcomes at baseline and at 6 and 12 weeks.

Outcome and group			Baseline (self-report)		1-6 weeks (sensor data)		7-12 weeks (sensor data)		1-12 weeks (sensor data)
**Weighing 5 days or more in a week, n (%)**									
	IG^a^ (n=15)	6 (40)		13 (87)		11 (73)		12 (80)	
	CG^b^ (n=17)	10 (53)		12 (71)		8 (47)		8 (47)	
	All (N=32)	16 (50)		25 (78)		19 (5)		20 (63)	
**Days with weighing (maximum: 42 in 6 weeks; 84 in 12 weeks), mean (SD); median**									
	IG (n=15)	N/A^c^		35.8 (6.0); 37		32.9 (10.5); 35		69.7 (16.1); 76	
	CG (n=17)	N/A		32.2 (10.2); 34		28.0 (10.9); 30.4		60.1 (18.7); 64	
	All (N=32)	N/A		34 (8.4); 37		30 (10.9); 31		64 (17.8); 64	
**Physical activity steps, mean (SD); median**									
	IG (n=15)	N/A		2742 (2499); 1986		3365 (2821); 2281		2887 (2821); 2133	
	CG (n=16)	N/A		2638 (1573); 2522		2444 (1757); 2244		2541 (1604); 2601	
	All (N=31)	N/A		2690 (2060); 2480		2737 (2596); 2262		2713 (2270); 2482	

^a^IG: intervention group.

^b^CG: control group.

^c^N/A: not applicable.

Overall, across the 2 groups, the female participants had lower trends in weight monitoring than the male participants (60 vs 70 days, respectively, out of 84); however, both biological sex groups had higher trends in the IG (men, 80; women, 61) compared with the CG (men, 62; women, 57) for days with weight monitoring.

#### Physical Activity

Trends in average physical activity steps from the 6th *week* to the 12th week were seen with modest increases in the IG and modest decreases in the CG (Table 4).

#### Multiple Behavior Engagement

**Figure 5:**
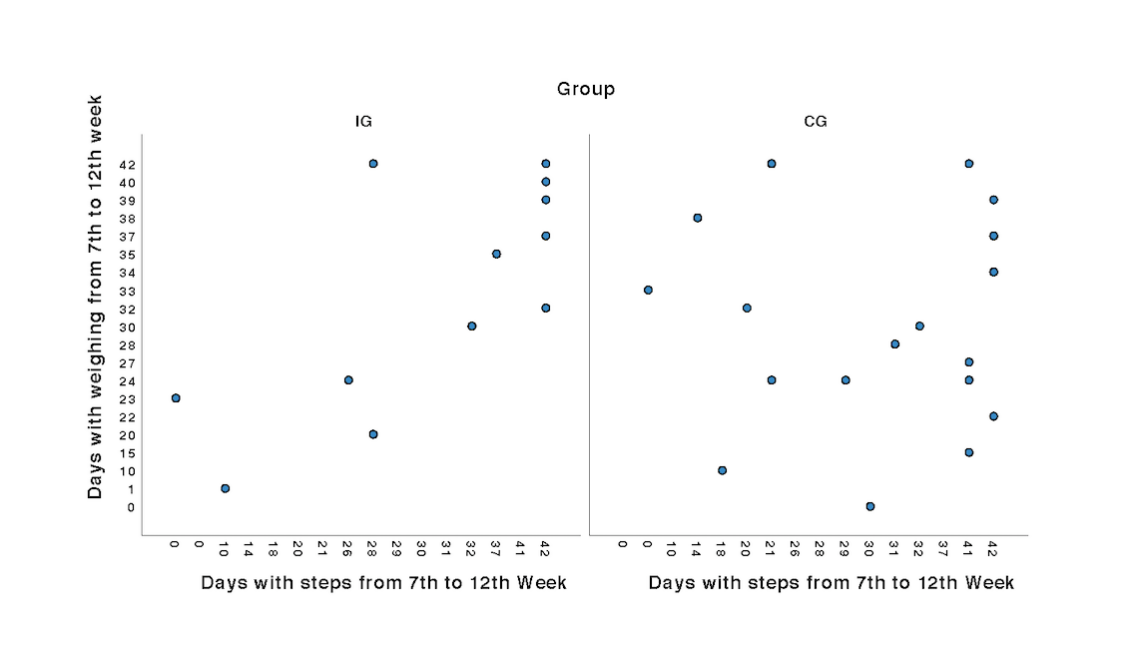
Engagement in dual behaviors of weight monitoring and physical activity. CG: control group; IG: intervention group.

HF self-management requires simultaneous engagement in multiple behaviors over an extended duration. The IG participants showed higher correlation (*r*=0.78; *P<*.001) between engaging in weight monitoring behaviors and engagement in physical activity behaviors from the 6th week to the 12th week than the CG participants (*r*=−0.01; *P=*.79; Figure 5).

#### HF Functional Status

At the end of 6 weeks, the IG demonstrated a clinically significant increase of 7 points on the KCCQ [50,61]. At the end of 24 weeks, both groups retained a clinically and statistically significant increase in functional status over baseline levels (Table 5).

#### Quality of Life

Both groups demonstrated a clinically [62] and statistically significant within-group increase in quality of life at 6, 12, and 24 weeks compared with baseline on the KCCQ (Table 5).

#### HF Self-management Knowledge

Both groups demonstrated modest but statistically significant within-group improvement in knowledge at 6, 12, and 24 weeks compared with baseline (Table 5).

#### Self-reported HF Self-management Behaviors

The IG participants consistently demonstrated improvement in self-reported self-management behaviors compared with baseline and retained a statistically significant improvement at the end of 24 weeks. Self-reported behaviors in the CG participants changed minimally in comparison with baseline (Table 5).

#### HF Self-efficacy

Both groups demonstrated within-group improvement in self-efficacy across the different time points, although the CG participants demonstrated higher trends of improvement (Table 5).

#### Motivation for HF Self-management Behaviors

The changes in autonomous regulation of behaviors were nonsignificant in both the groups at 6, 12, and 24 weeks (Table 5).

#### HF Hospitalization

Decreases in 1-month and 6-month hospitalization rates were seen in both the IG and CG, albeit tempered by the worldwide COVID-19 effect of decrease in HF hospitalizations [63,64] (Table 5).

**Table 5:** Survey outcomes at baseline and at 6, 12, and 24 weeks (N=38).

Paired outcome and group						Baseline			From baseline to 6 weeks								From baseline to 12 weeks								From baseline to 24 weeks			
						Value			Value				*P* value			Value					*P* value			Value			*P* value	
**HF^a^-related functional status**																												
		Population, n			N/A^b^			30				N/A			30					N/A			26			N/A		
		IG^c^, mean (SD)			66.6 (28.4)			6.9 (14.1)				.048^c^			3.7 (14.1)					.27			7.4 (10.7)			.07		
		CG^d^, mean (SD)			69.2 (20.8) 72.9			1.7 (11.8)				.54			4.1 (12.6)					.18			6.5 (11.7)			.03^e^		
**Quality of life**																												
	Population, n					N/A				30				N/A			30					N/A			26			N/A
	IG, mean (SD)					62.03 (24.1)				5.8 (13.1)				.07			8.2 (13.5)					.02^e^			10.8 (16.9)			.01^e^
	CG, mean (SD)					60.2 (20.7)			8.7 (15.3)				.02^e^			13.0 (19.3)					.01^e^			15.4 (19.6)			.003^e^	
**HF** **self-management knowledge**																												
	Population, n					N/A					30			N/A				30				N/A			26			N/A
	IG, mean (SD)					24.5 (2.6)					1.9 (2.5)			.003^e^				1.4 (1.7)				.002^e^			1.1 (2.1)			.03^e^
	CG, mean (SD)					25.0 (2.4)					2.1 (2.5)			.001^e^				1.1 (1.5)				.005^e^			1.1 (1.7)			.01^e^
**Self-reported** **HF** **self-management behaviors**																												
	Population, n					N/A					30			N/A				31				N/A			25			N/A
	IG, mean (SD)					74.1 (21.1)					4.9 (20.4)			.31				9.3 (16.5)				.02^e^			10.2 (13.4)			.004^e^
	CG, mean (SD)					81.7 (15.5)					−2.7 (9.9)			.26				2.6 (12.4)				.4			0.5 (9.8)			.8
**HF** **self-efficacy**																												
			Population, n			N/A			30				N/A			31					N/A			25			N/A	
			IG, mean (SD)			69.9 (20.7)			2.2 (25.7)				.7			1.0 (20.5)					.8			3.0 (18.0)			.5	
			CG, mean (SD)			62.6 (18.4)			6.9 (18.1)				.11			10.2 (12.3)					.002^b^			7.0 (16.5)			.08	
**Motivation for** **HF** **self-management behaviors**																												
		Population, n			N/A			30				N/A			30					N/A			25			N/A		
		IG, mean (SD)			1.63 (2.2)			−0.1 (1.2)				.75			0.1 (2.6)					.9			−0.6 (2.5)			.4		
		CG, mean (SD)			1.64 (1.6)			−0.2 (2.2)				.8			–0.3 (3.0)					.7			−0.2 (2.7)			.8		
**HF** **hospitalization**																												
	**In the last month**																											
				Population, n		N/A	31						N/A						31		N/A			26			N/A	
				IG, mean (SD)		5 (26.3)	1 (7)						N/A						0 (0)		N/A			1 (7)			N/A	
				CG, mean (SD)		6 (31.6)	1 (6)						N/A						1 (6)		N/A			0 (0)			N/A	
	**In the past 6 months**																											
				Population, n		N/A			N/A				N/A			N/A					N/A			N/A			N/A	
				IG, mean (SD)		12 (63)			N/A				N/A			N/A					N/A			2 (15)			N/A	
				CG, mean (SD)		9 (47)			N/A				N/A			N/A					N/A			2 (15)			N/A	

^a^HF: heart failure.

^b^N/A: not applicable.

^c^IG: intervention group.

^d^CG: control group.

^e^Significant at *P*<.05.

## Discussion

### Principal Findings

We have demonstrated the feasibility of conducting a randomized controlled trial to compare an SCDG intervention and a sensors-only intervention for improving older adults’ HF self-management behaviors of daily weight monitoring and physical activity and other health outcomes. Despite the constraints imposed by the COVID-19 pandemic, we successfully attained benchmark parameters for the feasibility outcomes of recruitment, retention, intervention engagement, and satisfaction with the intervention among older adults diagnosed with HF. The advantages of recruiting participants and conducting the trial remotely enabled us to increase access to our study from 2 counties to more than 18 counties, which included rural areas. The older adult participants in our study were able to use the digital game app and sensors regardless of their education level, familiarity with digital games, or smartphone platform (ie, iOS or Android).

Regarding the behavior outcomes, the IG demonstrated better trends in the primary outcome of weight monitoring behavior, with a higher average of weight monitoring days than the CG: 46% more IG participants than CG participants attained the clinically significant level of weighing 5 days or more in a week. Similarly, the IG participants demonstrated higher engagement in the dual behaviors of weight monitoring and physical activity than the CG participants. Moreover, the IG participants demonstrated significant within-group improvement in self-reported HF self-management behaviors (weight monitoring, exercise, salt restriction, medication adherence, crisis recognition, and related follow-up), which persisted at 24 weeks. This result is promising because improving overall HF outcomes depends on engagement in multiple HF self-management behaviors over long time intervals.

Ours is one of the few studies to capture daily physical activity steps of older adults with HF over 12 weeks. Although both IG and CG participants demonstrated an increase in average daily physical steps from the 6th week to the 12th week, the increase was still lower overall than recommended for physical activity steps [65]. Given the technical issues with an outdated tracker, the SCDG may motivate higher increases in step levels with better trackers. Although typical recommended levels for cardiovascular health benefits have ranged from 10,000 [65] to 15,000 [66] steps a day, a study with 16,000 older women found that even an average of 4400 steps a day resulted in significantly lower mortality rates [67]. However, older adults with HF often suffer from frailty and fatigue; therefore, it may be harder for them to reach the optimal step levels. In a recent study that examined the walking activity of adults with HF using a step counter over a year, younger age, higher ejection fraction, and lower HF classification were found to be significantly correlated with the number of daily steps [68]. Whether modest increases in steps result in health benefits among older adults with HF remains to be seen and may signify a need for additional supportive or palliative interventions that relieve physical or mental symptom burden to ensure optimal quality of life among patients with HF [69]. Nevertheless, studies like ours demonstrate actual physical activity engagement in real-world situations and examine the longitudinal relationship between clinically significant physical activity steps and optimal HF outcomes. A recent position paper on measuring physical activity by varied activity monitors for HF in real-world conditions has provided key criteria for the selection of activity monitors based on the aims of the research and observation metrics [70]. On the basis of our participants’ experience with the SCDG, future iterations could include the ability to set realistic step goal levels for participants with HF that are lower than 3000 steps and game play duration that is longer than 12 weeks.

The within-group trends of improvement in functional status and quality of life in both the IG and CG that persisted until the end of the study demonstrate the potential of digital health interventions to improve outcomes among older adults with HF. Similarly, trends of improvement in HF self-efficacy were observed in both the IG and CG. Both groups were allowed to retain the sensor devices after 12 weeks of behavior data collection, which might have contributed to persistent improvement in perceived functional status, quality of life, and self-efficacy in comparison with baseline levels. The lack of perceptible changes in autonomous regulation of behaviors in either group could be explained by the participants’ perceiving the SCDG and the sensors-only interventions as expecting accountability of their behaviors, which may be analogous to controlled regulation of behavior. Future research can help explore the need and preferences for either kind of behavior regulation for sustaining HF self-management behaviors.

Improved HF management at the clinical level may have resulted in the baseline knowledge of HF self-management being already high in both groups. Still, both groups demonstrated trends of modest improvements in knowledge at 6 and 12 weeks that were maintained at 24 weeks.

Reduction in HF hospitalization is the desired distal outcome of improved self-management behaviors. Although both groups demonstrated reduction in hospitalization, it is unclear whether the reduction resulted from the interventions or from the worldwide reduction in cardiac-related hospitalizations due to the COVID-19 pandemic [62,64]. The reduction in hospitalizations by more than 30% in both the groups persisted until the end of the 24-week study period.

### Comparison With Prior Work

Recent studies have explored the feasibility of presenting data from fitness tracker sensors or Bluetooth-enabled weighing scales to track behaviors and provide feedback to patients with HF through an mHealth app. In a single-group study with 20 individuals with HF who were followed over 6 months to track weight monitoring, physical activity, and medication adherence, 60% of the participants wore trackers and 45% used the weighing scale for more than 70% of the days [71]. In our study, among the 32 participants who installed the SCDG app, 27 (80%) wore the trackers for more than 70% of the days and used the weighing scale for more than 80% of the days at the end of 12 weeks. In addition, the IG participants in our study experienced an increase in HF functional status and similar increases in knowledge in comparison with the IG participants in another recent study with an mHealth app intervention for HF that also employed a Bluetooth weighing scale [72]. Although our study’s results with the SCDG app compare favorably with those of similar studies that have examined mHealth apps and sensor devices with patients with HF, given the small sample size in our feasibility trial, the results and findings from our trends analysis should be treated with caution.

### Limitations

Digital literacy is often a concern when digital health interventions are implemented with older adults [73]. Our study team’s prior experience in conducting usability assessments with older adults with HF as well as in installation of, and training for, devices and apps at the participants’ homes [25] helped us to design the information and content to support the participants’ self-installation of the apps and use of the devices. Nevertheless, the study might have suffered from selection bias because participation was voluntary and participants already comfortable with using technology might have been especially motivated to participate. In addition, despite block randomization and stratification by biological sex, a higher proportion of participants in the IG were married or had a partner. Having a partner to support self-management efforts might have resulted in better trends of behavior outcomes in the IG.

Another limitation was the research team’s inability to intervene immediately in the event of missing behavioral data or unsafe weight changes throughout the 12-week game play duration. Our approach of using the Twilio app (Twilio Inc) [74] for alerting the nursing research assistant to the absence of physical activity or weight monitoring data or to sudden weight changes became unmanageable because of the high volume of text messages. In future studies, developing a dashboard that will allow easier visualization of behavioral data from all participants will provide us with the opportunity to intervene immediately in the event of absent or unsafe levels of behavioral data.

### Conclusions

This pilot randomized controlled trial indicates that playing an SCDG app on smartphones is feasible and acceptable for older adults with HF for motivating daily weight monitoring and physical activity. The participants who played the SCDG demonstrated positive trends in daily weight monitoring and physical activity behaviors and reduction in HF hospitalization compared with the sensors-only group. This study justifies fully powered efficacy trials with digital gaming solutions to motivate adherence to HF self-management behaviors and improve the health outcomes of older adults with HF.

## Appendix

Multimedia Appendix 1 CONSORT-eHEALTH checklist (V 1.6.1).

## Abbreviations

CG: control groupCONSORT: Consolidated Standards of Reporting Trials
HF: heart failure
IG: intervention group
KCCQ: Kansas City Cardiomyopathy Questionnaire
mHealth: mobile health
SCDG: sensor-controlled digital game
